# A sticky situation: a case of *Actinomyces viscosus* vertebral osteomyelitis

**DOI:** 10.5194/jbji-6-39-2020

**Published:** 2020-09-09

**Authors:** Stephanie L. Grach, Aaron J. Tande

**Affiliations:** 1 Department of Internal Medicine, Mayo Clinic, Rochester, 55905, USA; 2 Division of Infectious Diseases, Mayo Clinic, Rochester, 55905, USA

## Abstract

*Actinomyces viscosus* is an oral bacterium that is rarely virulent in humans, with most case presentations involving dental and maxillofacial infections. We describe the first reported case of *A. viscosus* vertebral osteomyelitis in a patient who had a significant response to penicillin after minimal response to cephalosporin therapy.

## 1 Introduction

*Actinomyces viscosus* is an anaerobic gram positive bacillus that colonizes the oral cavity in up to 70 % of individuals, with few instances of pathogenicity outside of dental cases (Eng et al., 1981). Osteomyelitis as a manifestation of *A. viscosus* infection has been reported in the mandible and rib; cases have also been reported in the spines of animals (Price et al., 1982; Thadepalli and Rao, 1979; Johnson et al., 1984). This clinical vignette represents the first reported case of *A. viscosus*-associated vertebral osteomyelitis in humans.

## 2 Case description

**Figure 1:**
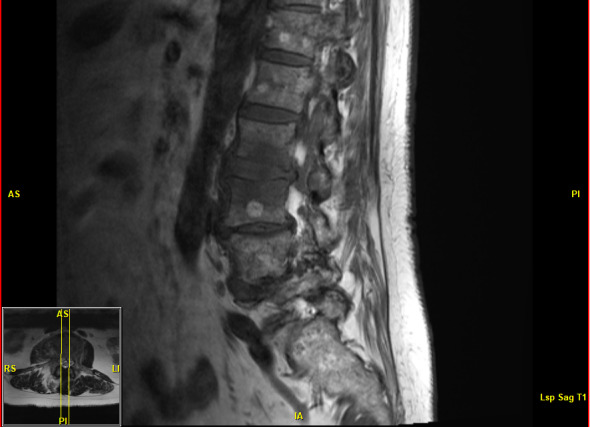
MRI demonstrating abnormal T2 hyperintensity with accompanying inflammatory changes at the L2–L3 level. Chronic multilevel severe spinal canal stenosis is also present.

A 76-year old immunocompetent female retired physical therapist with a medical history of hypertriglyceridemia, basal cell carcinoma, severe lumbar stenosis, and left total knee arthroplasty presented to the primary care office with lower back pain and right leg radiculopathy. She had noted acute onset of lower back pain while at her local shopping center. The pain radiated into the buttocks and posterior thigh and was associated with intermittent spasms which limited her movement. She did not note any fever, chills, motor weakness, sensory loss, or change in bowel or bladder function. Her primary care physician ordered magnetic resonance imaging (MRI) which demonstrated L2–L3 disk space infection, namely abnormal T2 hyperintensity in the disk space, abnormal marrow edema in the adjacent endplates with minimal destruction of the inferior endplate of L2, and accompanying inflammatory phlegmon extending laterally to the right greater than left psoas muscles; no discrete abscess was identified (Fig. 1). Based on these radiological findings, she was instructed to seek admission to the hospital for vertebral osteomyelitis. Orthopedic Infectious Disease was consulted for further workup and management. On presentation to the hospital, the patient was hemodynamically stable with negative review of symptoms except as above.

Physical exam was only notable for mild percussion tenderness over the L2–L3 area. Initial laboratory studies included an elevated erythrocyte sedimentation rate (ESR) at 62 mm h${}^{-1}$ and C-reactive protein (CRP) at 50.1 mg L${}^{-1}$. White blood cell count (WBC) was normal at $9.4\times 10^{9}$ cells L${}^{-1}$. Blood cultures were obtained, and based on the MRI and elevated inflammatory markers, a CT-guided biopsy was performed. Following the biopsy, she was started on empiric IV vancomycin and IV ceftriaxone. Blood cultures grew two of three bottles positive for *Actinomyces viscosus* after 51 h, susceptible to penicillin <0.5 mcg mL${}^{-1}$ and clindamycin <2 mcg mL${}^{-1}$. Biopsy cultures at the L2–L3 levels of the spine demonstrated growth of *Actinomyces viscosus* on aerobe/anaerobe bacterial culture testing, susceptible to penicillin <0.5 mcg mL${}^{-1}$, piperacillin/tazobactam <32/4 mcg mL${}^{-1}$, ertapenem <4 mcg mL${}^{-1}$, clindamycin <2 mcg mL${}^{-1}$, moxifloxacin <2 mcg mL${}^{-1}$, and minocycline <4 mcg mL${}^{-1}$ and resistant to metronidazole >16 mcg mL${}^{-1}$. Gram stain testing was negative for growth. Upon further review with the patient after discovery of the oral pathogen, it was revealed that she had a dental implant infection 4 years prior to her osteomyelitis presentation, though she had no known complications or further symptoms in the meantime; a routine dental exam earlier in the year also had no associated abnormal findings. A PICC line was placed with subsequent cultures negative for bacterial growth, and the patient was discharged on 2 g IV ceftriaxone q24 hours with a plan for 6 weeks of therapy.

On treatment day 35 of 42, the patient presented to the Infectious Disease clinic with increasing lower back pain and right radiculopathy as well as new erythematous rash on the proximal forearms. Repeat labs showed an increase in CRP to 89 mg L${}^{-1}$, and repeat MRI demonstrated only minimal soft tissue response to therapy. Given a worsening clinical picture with rising CRP and increasing pain, arrangements were made to start IV penicillin G at 20 000 000 U q24 hours via continuous infusion. The patient was seen 2 weeks later in the ID clinic with drastic clinical improvement; ESR and CRP were now normal at 27 mm h${}^{-1}$ and 3 mg L${}^{-1}$, respectively. She completed 4 weeks of IV penicillin G and was transitioned to oral penicillin VK 1 g three times daily (tid) ×18 weeks with full resolution of her infection and reported radicular back pain. At the conclusion of her treatment, physical examination demonstrated no focal tenderness or pain with movement, and all lab values were within normal range.

## 3 Discussion

**Figure 2:**
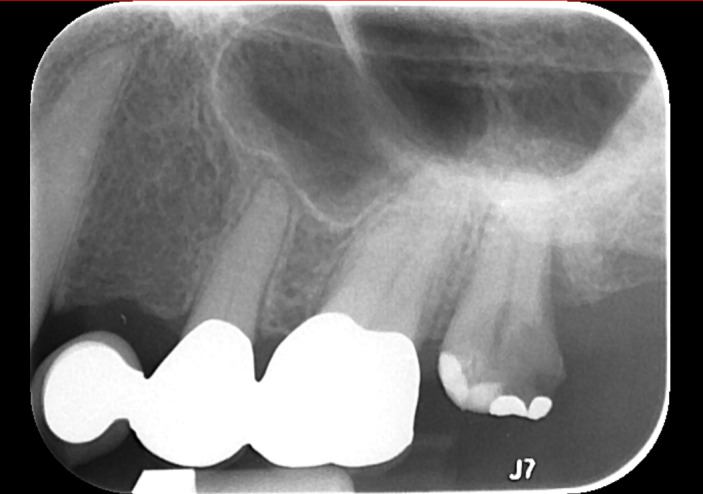
Periapical radiograph demonstrating a large carious lesion at no. 15 with associated widening of periodontal ligament and periapical radiolucency consistent with implant infection.

The *Actinomyces* spp. consists of filamentous, anaerobic gram-positive bacilli that may colonize the oral, gastrointestinal, and urogenital tracts in most humans. Most forms of *Actinomyces*, including viscosus, are facultative anaerobes. Actinomycosis refers to the state of invasive bacterial disease by one of the various species, which may vary based on the specific organism and its location. *Actinomyces israelii* is the most common implicated pathogen in actinomycosis, especially with respect to craniofacial involvement, followed by *Actinomyces meyeri*, *A. viscosus*, and other species (Valour et al., 2014). *A. viscosus*, named for its colonies being “sticky” in nature when grown, is an oral pathobiont which rarely causes infection in humans outside of the oropharyngeal cavity, though cases have been reported as included in this discussion. Osteomyelitis is an exceptionally rare presentation of *Actinomyces viscosus* outside of animal populations, with no instance of vertebral osteomyelitis previously reported in humans (Price et al., 1982; Thadepalli and Rao, 1979; Johnson et al., 1984). *A. visocus* is not uncommon in dental infections and is known to colonize by supragingival plaque and root surfaces, triggering periodontal inflammation via lipoprotein activation of toll-like receptor 2 (Shimada et al., 2012). Our patient did have a history of dental implant infection 4 years prior, though the culprit organism was not identified, and there was no evidence of persistence at subsequent dental examinations (Fig. 2). It is difficult to conclude whether this was therefore related to her presenting osteomyelitis. She also did not demonstrate clinical signs of endocarditis, and an echocardiogram was not performed, though this could be evaluated for similar cases if the concern is present. Manifestations of actinomycosis can be quite severe, including those caused by *A. viscosus*, as evidenced by the significant L2–L3 disk space involvement in our case, and regardless of the site may require months to a year of penicillin or similar antibiotic therapy (Valour et al., 2014).

This report history is most notable for our patient's dramatic improvement with penicillin. Ceftriaxone is often used during outpatient antibiotic therapy given the favorable once daily dosing structure and has been successfully used in cases of *A. viscosus* endocarditis (Hamed, 1998). However, despite the efficacy of ceftriaxone in cases of endocarditis and more common species of *Actinomyces*, *A. viscosus* has most dependably responded to penicillin in the literature across all sites, as was the case with our patient's infection (Eng et al., 1981; Smith et al., 2005; Scarano et al., 1999). This was notably demonstrated in a case of prosthetic hip joint infection (Cohen et al., 1993). This dependable response to penicillin is critical knowledge for severe cases of *Actinomyces viscosus*, as disseminated infection or opportunistic involvement in transplant and other immunocompromised patients may present significant danger if left treated ineffectively (Vega et al., 2017; Habib et al., 2018). In our case, ceftriaxone was chosen for its more favorable dosing and also due to insurance concerns related to coverage of a pump for IV penicillin. Unfortunately, the patient experienced almost 6 weeks of clinically apparent persistent infection while on cephalosporin therapy; this caused significant lifestyle interruption that would not have otherwise occurred had she been initiated on penicillin upon hospital discharge. Pump arrangements were able to be made upon failed response to ceftriaxone. High-dose IV penicillin at 20 million units was subsequently started as manifestations of actinomycosis have been shown to be responsive to 18–24 MIU (Wong et al., 2011).

Given these considerations, it may be prudent to pursue high-dose penicillin therapy from the start in patients suffering from vertebral osteomyelitis secondary to *A. viscosus*. For milder cases amenable to oral penicillin therapy or on transition off of IV therapy as we had done with oral penicillin VK, amoxicillin has been demonstrated to be an effective agent in cases of other cases of actinomycosis (Valour et al., 2014). In addition to ceftriaxone, clindamycin, doxycycline, and moxifloxacin have also demonstrated in vitro activity against *A. viscosus* and may be explored as alternative agents in penicillin-allergic individuals (LeCorn et al., 2007).

## 4 Conclusions

*Actinomyces viscosus* is primarily associated with oral infections and colonization; this case represents the first reported instance of this particular species causing vertebral osteomyelitis. Although cephalosporin therapy has traditionally been effective in treating members of the *Actinomyces* genus, there are limited data to support ceftriaxone for serious *Actinomyces* infection in the setting of osteomyelitis. Going forward, we recommend that penicillin be considered first-line therapy for vertebral osteomyelitis associated with the *Actinomyces* species.

## Data Availability

Data are not available in a public data repository but are available upon request from the authors.
